# Role of *p63* and *p73* isoforms on the cell death in patients with hepatocellular carcinoma submitted to orthotopic liver transplantation

**DOI:** 10.1371/journal.pone.0174326

**Published:** 2017-03-28

**Authors:** Raúl González, Ángel J. De la Rosa, Alessandro Rufini, María A. Rodríguez-Hernández, Elena Navarro-Villarán, Trinidad Marchal, Sheila Pereira, Manuel De la Mata, Martina Müller-Schilling, Juan M. Pascasio-Acevedo, María T. Ferrer-Ríos, Miguel A. Gómez-Bravo, Francisco J. Padillo, Jordi Muntané

**Affiliations:** 1 Institute of Biomedicine of Seville (IBiS), Hospital University “Virgen del Rocío”/IBiS/CSIC/University of Seville, Seville, Spain; 2 Department of Cancer Studies, CRUK Leicester Cancer, Leicester, United Kingdom; 3 Pathology Department, IMIBIC/Hospital University “Reina Sofía”, Córdoba, Spain; 4 Gastroenterology Department, IMIBIC/Hospital University “Reina Sofía”, Córdoba, Spain; 5 Centro de Investigación Biomédica en Red de Enfermedades Hepáticas y Digestivas (CIBERehd), Madrid, Spain; 6 Gastroenterology and Hepatology Department of Internal Medicine IV, University Hospital Heidelberg, Heidelberg, Germany; 7 Gastroenterology Department, Hospital University “Virgen del Rocío”/IBiS/CSIC/University of Seville, Seville, Spain; 8 Department of General Surgery, Hospital University “Virgen del Rocío”/IBiS/CSIC/University of Seville, Seville, Spain; University of Navarra School of Medicine and Center for Applied Medical Research (CIMA), SPAIN

## Abstract

**Background & Aims:**

Patients with hepatocellular carcinoma (HCC) submitted to orthotopic liver transplantation (OLT) have a variable 5-year survival rate limited mostly by tumor recurrence. The etiology, age, sex, alcohol, Child-Pugh, and the immunesuppressor have been associated with tumour recurrence. The expression of ΔNp73 is related to the reduced survival of patients with HCC. The study evaluated the expression of p63 and p73 isoforms and cell death receptors, and their relation to tumour recurrence and survival. The results were *in vitro* validated in HCC cell lines.

**Methods:**

HCC sections from patients submitted to OLT were used. The *in vitro* study was done in differentiated hepatitis B virus (HBV)-expressing Hep3B and control HepG2 cells. The expression of cell death receptors and cFLIP_S/L_, caspase-8 and -3 activities, and cell proliferation were determined in control and p63 and p73 overexpressing HCC cells.

**Results:**

The reduced tumor expression of cell death receptors and TAp63 and TAp73, and increased ΔNp63 and ΔNp73 expression were associated with tumor recurrence and reduced survival. The *in vitro* study demonstrated that HBV-expressing Hep3B vs HepG2 cells showed reduced expression of p63 and p73, cell death receptors and caspase activation, and increased cFLIP_L_/cFLIP_S_ ratio. The overexpression of TAp63 and TAp73 exerted a more potent pro-apoptotic and anti-proliferative effects in Hep3B than HepG2-transfected cells which was related to cFLIP_L_ upregulation.

**Conclusions:**

The reduction of TAp63 and TAp73 isoforms, rather than alteration of ΔN isoform expression, exerted a significant functional repercussion on cell death and proliferation in HBV-expressing HepB cells.

## Introduction

Hepatocellular carcinoma (HCC) is the fifth most common neoplasia in the world, and the third most common cause of cancer-related mortality worldwide (600,000 deaths per year) [1, 2]. Patients with one single nodule (≤5 cm) or three smaller nodules (≤3 cm), that present portal hypertension, bilirubinemia and associated diseases are recommended for orthotopic liver transplantation (OLT). HCC patients subject to OLT following strictly the Milan’s criteria have a high survival rate (60–80%) and suffer low rates of tumor recurrence (10–20%) at 5-years post-surgery [3].

A meta-analysis of gene expression profiles identified three robust HCC subclasses (termed S1, S2, and S3), characterized by an aberrant activation of the TGF-β and Wnt/β-catenin signaling pathways (S1), Myc and Akt activation, as well as down-regulation of IFN-target genes (S2), and activation of p53 and p21 target genes, and genes associated with hepatocyte differentiation (S3) [4]. In this context, reduced expression of p53 in HCC was related to the most aggressive S1 and S2 subclasses [5].

The transcription factor p53 regulates many targets genes related to cell cycle arrest, apoptosis, DNA repair or metabolism, typically in response to stress signals, caused for example by oncogenic alterations. Consequently, p53 is known to exert oncosuppressor functions [6, 7] and the p53 pathway is inactivated in the majority of human malignancies [8]. The identification and characterization of the p53/p63/p73 network provides evidence of a tight link between developmental processes and tumorigenesis [9]. Although p63 and p73 are both homologs of p53, they are encoded by different genes and generate a complex variety of protein isoforms following the use of two alternative gene promoters, and extensive differential splicing of the primary transcript. Hence, p63 and p73 share some p53 functions, such as cell cycle arrest and apoptosis, but the presence of sterile α motif at the COOH-terminus of the TAp63 and TAp73 isoforms suggest that they may be more involved than p53 in developmental regulation [9]. The isoforms encoded from the upstream promoter contain the conserved N-terminal TA domain which is responsible for the full pro-apoptotic and cell cycle arrest properties of both TAp63 and TAp73. However, the isoforms encoded from the downstream promoter lack the entire TA domain, thus generating the dominant-negative or ΔN isoforms that counteract the transactivation activities of the TA isoforms. Like p53, TAp63 and TAp73 activate genes regulating different steps of the apoptotic program [10]. p53 mediates the induction of CD95 expression in response to anticancer DNA-damaging drugs in hepatoma, gastric cancer, colon cancer, and breast cancer cell lines [11]. Bleomycin and doxorubicin increased cell death receptor expression in p53-wild type HepG2 and in p53-deficient Hep3B cells suggesting that p53, TAp63 and TAp73 can trigger expression of cell death receptors and consequently sensitize HCC cells toward apoptosis induced by DNA-damaging drugs [12].

The aim of this study was to characterize the pattern of expression of TAp63, ΔNp63, TAp73 and ΔNp73 isoforms, and cell death receptors in HCC from patients submitted to OLT, and to correlate their expression with clinical data such as tumor recurrence and overall survival. The reduced survival of patients by tumor recurrence was associated with increased ΔNp63 and ΔNp73 expression, as well as reduction of TAp63 and TAp73 and cell death receptor expression. Moreover, p63 and p73 status dictated survival selectively in patients bearing HBV infection, but not hepatitis C virus (HCV) and alcohol etiologies. The *in vitro* study showed a reduced expression of p63 and p73 isoforms and cell death receptors, and caspase-3 and -8 activities in Hep3B vs HepG2. Although the overexpression of TAp63 and TAp73 induced a stronger effect on cell death receptor upregulation in HepG2 than Hep3B cells, the latest cell line showed more potent pro-apoptotic and anti-proliferative activities, associated with cFLIP_L_ upregulation. The overexpression of ΔNp63 and ΔNp73 exerted a downregulation of TNF-R1 expression in HepG2 cells.

## Material and methods

### Cell line culture

Human hepatoma cell lines, HepG2 and Hep3B obtained from the American Type Culture Collection (ATCC-LGC Standards). Hep3B cells express the two major polypeptides of the HBV surface antigens, providing an excellent experimental model for investigating its role [13]. Cells were routinely maintained in Minimum Essential Medium with Earle’s salts at pH 7.4, 5% CO_2_ in air at 37°C supplemented with 2 mM L-glutamine, 0.1 mM non-essential amino acids, 1.0 mM sodium pyruvate, 100 U/mL penicillin, 100 μg/mL streptomycin, and 10% non-heat-inactivated fetal bovine serum.

### Overexpressing plasmid of TAp63α, ΔNp63α, TAp73α and ΔNp73α

The cDNA of TAp63α-HA-tagged, ΔNp63α-HA-tagged, TAp73α-HA-tagged and ΔNp73α-HA-tagged were cloned in the pcDNA 3.1 vector. The pcDNA 3.1 empty vector was used as a control. The overexpression was performed pre-incubating hepatocyte jetPEITM transfection reagent (Polyplus-Transfection SA, Illkirch France) with plasmids for 20 min at room temperature. This mixture was transferred to the culture medium without fetal bovine serum and antibiotics for 6 hours. The study was initiated 18 hours after to allow plasmid full expression, and variables were assessed 48 hours after.

### Patients

Tumor liver sections from patients (n = 74, 64 men and 10 women) who underwent OLT for HCC were collected from June 1992 to December 2015 from two different cohorts obtained in the Hospital University “Reina Sofía” (Córdoba, Spain) and the Hospital University “Virgen del Rocío” (Sevilla, Spain), with follow-up to 36 months. Eleven patients were excluded of the whole cohort by unknown etiology (2 women, 1 men), unknown HCC staging (2 women, 2 men), as well as combined etiologies HCV+alcohol (2 men) and HBV+alcohol (2 men). The grade of HCC differentiation was estimated according to the Edmondson-Steiner criteria that considered the characteristics, such as size, morphology, and mitotic figures, and grouped in the present study as well-differentiated (grade I, n = 22) and moderately-poorly differentiated (grade II-IV, n = 41). The etiology of the patients was HCV (n = 24, 38%) HBV (n = 19, 30%), and alcohol-associated cirrhosis (n = 20, 32%). The causes of death during follow-up were tumor recurrence or other causes such as pneumonia, fungal sepsis, heart complications and septic shock. Additional demographic, clinical and pathological features of the patients are showed in Table 1. The procedures of managing human samples and personal data were in accordance with the ethical standards of the responsible committee on human experimentation and with the Helsinki declaration of 1975, as revised in 1983. The Ethical and Research Committees of the Hospital University “Reina Sofía” (Córdoba, Spain), the Ethical Committee of the Hospital University “Virgen del Rocío” (Sevilla, Spain), and the Regional Biobank of Andalusian Public Health System (Sevilla, Spain) that coordinates the activity of the local biobank in the Hospital University “Reina Sofía” (Córdoba, Spain) and the Hospital University “Virgen del Rocío” (Sevilla, Spain) reviewed and approved the study, and authorized the use of liver biopsies from the patients with HCC submitted to OLT to be included in the present study.

**Table 1:** Clinical and pathological characteristics of the patients.

	Well-differentiated HCC (Grade I) (n = 22)	Moderately-Poorly differentiated HCC (Grade II-IV) (n = 41)
**Sex**		
Male	22 (100%)	35 (85%)
Female	0 (0%)	6 (15%)
**Tumor size**		
< 5 cm	16 (73%)	37 (90%)
5 cm <	0 (0%)	4 (10%)
**Number of foci**		
Uninodular (1)	11 (50%)	27 (66%)
Multinodular (2)	6 (27%)	10 (24%)
Multinodular (3–4)	5 (23%)	4 (10%)
**Etiology**		
HCV (Alive,)	8 (36%)	12 (30%)
HCV (Death HCC recurrence)	1 (5%)	1 (2%)
HCV (Death other causes)	0 (0%)	2 (5%)
HBV (Alive)	4 (20%)	9 (23%)
HBV (Death HCC recurrence)	1 (5%)	1 (2%)
HBV (Death other causes)	1 (5%)	2 (5%)
Alcohol (Alive)	6 (29%)	8 (21%)
Alcohol (Death HCC recurrence)	0 (0%)	0 (0%)
Alcohol (Death other causes)	1 (5%)	5 (12%)

HCC: hepatocarcinoma; HBV: Hepatitis B virus; HCV: Hepatitis C virus

### Detection of TAp63, ΔNp63, TAp73, ΔNp73, TNF-R1, CD95, TRAIL-R1 and cFLIP_L_/_S_ protein expression in liver sections and cell lysate

The expression of p53 isoforms and cell death receptors were analyzed in HCC tissue by immunohistochemistry, and by Western-Blot analysis in cell lysate [14] or in cytoplasmic/nuclear [15] fractions. Deparaffinized tissue sections (5 μm) were hydrated, blocked specific sites, and incubated with primary antibodies against TNF-R1 (sc-7895, Santa Cruz Biotechnology, Santa Cruz, CA, USA), CD95 (sc-715), TRAIL-R1 (sc-6823), TAp63 (sc-8608) (TAp63α, TAp63β and TAp63γ), ΔNp63 (sc-71827) (ΔNp63α), TAp73 (Img-246, Imgenex, San Diego, CA, USA) (TAp73α, TAp73β and TAp73γ), and ΔNp73 (Img-313 A) (ΔNp73) overnight at room temperature in wet chamber. Sections were incubated with the corresponding fluorescent (Alexa 488) secondary antibodies (ThermoFisher Scientific, Waltham, MA USA) for 5 hours at room temperature. The contrasting nuclear staining was carried out with DRAQ-5th (Red Fluorescen Cell-Permeable DNA probe, Biostatus Limited, Shepshed, United Kingdom). The images were captured using an Olympus BX61 motorized upright microscope with fluorescence and phase contrast optics for immunofluorescence imaging, coupled to Application Suite Advanced Fluorescence and ImageJ software. The intensity of the signaling in the section stained with control isotype antibodies was used as background value of sections stained with the specific antibodies in each sample. The software allows to refer the value of intensity to the surface of tissue section in each sample.

The expression of TAp63, ΔNp63, TAp73 and ΔNp73 was assessed by Western-blot in cytoplasmic and nuclear fractions. Proteins (20–100 μg) were loaded and separated onto any-kD 18-well CriterionTM TGX Stain-FreeTM precast SDS-polyacrylamide gels (Bio-Rad Laboratories, Hercules, CA, USA) at 300 V 20–25 min, and proteins transferred to PVDF membrane. A stain-free blot image was taken using the ChemiDocTM MP System for protein measurement in each sample lane. Blots were incubated using commercial polyclonal primary antibodies described above, and with anti-cFLIP (1/1000) (Alx-804-428, Enzo Life Sciences, Plymouth, PA, USA), and followed of the corresponding secondary antibodies. The expression of β-actin (sc-47778) or histone H3 (Ref 4499, Cell Signaling Technology), the stain-free technology and ponceau staining were used as control of protein loading. Chemiluminiscence was measured using an Infinite 200 PRO Microplate Reader (TECAN Group Limited, Männedorf, Suiza).

### RNA extraction and reverse transcriptase-quantitative PCR for measuring TAp63α, ΔNp63α, TAp73α and ΔNp73α expression in HepG2 and Hep3B

RNA was isolated by TRIsure Reagent (BIO-38033, Bioline Reagents Ltd., London, United Kingdom), submitted to retrotranscription (Ref 4368813, Applied Biosystem, Foster City, CA, USA) and amplification by RT-qPCR (Ref 172–5121, iTaq Universal SYBR Green SuperMix, BioRad Laboratories). RT-PCR was performed in ViiA 7 Real Time PCR System (Thermo Fisher Scientific). Reactions were performed in 96-well plates with optical sealing tape (Applied Biosystem) in 20 μL total volume containing SYBR Green SuperMix and the corresponding cDNA. The conditions for amplification were as follows: denaturation step of 95°C for 40 sec, followed by 40 cycles of 95°C for 20s, 60°C for 60 sec, 95°C for 15 sec; with final elongation step of 95°C for 15 sec, 60°C for 60 sec and 95°C for 15 sec. All primers were designed using online Primer3 software Primer3input: TAp63α forward: 5´-AGTCCAGAGGTTTTCCAGCA-3´ and reverse: 5´- GAGGAGCCGTTCTGAATCTG-3´, ΔNp63α forward: 5´-tggagccagaagaaaggaca-3´ and reverse: 5´-GAGGAGCCGTTCTGAATCTG-3´ and TAp73α forward: 5´-CCTCTGGAGCTCTCTGGAAC-3´ and reverse: 5´-TGCTCAGCAGATTGAACTGC-3´ and ΔNp73α forward: 5´-AAGCGAAAATGCCAACAAAC-3´ and reverse: 5´-CACCGACGTACAGCATGGTA-3´. β-actin was used as internal control in parallel for each run: β-actin forward: 5´-GGACTTCGAGCAAGAGATGG-3´ and reverse 5´-AGCACTGTGTTGGCGTACAG-3. The Livak method was used to analyze the relative quantification RT-PCR data, in which absolute quantification determines the input copy number, usually by relating the PCR signal to a standard curve and relative quantification relates the PCR signal of the target transcript in a treatment group compared to untreated group [16]. This method is a suitable option to analyze the relative changes in gene expression from quantitative RT-PCR experiments.

### Measurement of apoptosis

Caspase-8 and -3-associated activities were determined following the indications of Caspase-Glo^®^ 8 (G8201, Promega, Fitchburg, Wisconsin, USA) and Caspase-Glo^®^ 3 (G8091). The luminescent signal was extrapolated into a calibration curve developed using recombinant purified enzyme. Chemiluminiscence was measured using an Infinite 200 PRO Microplate Reader (TECAN).

### Cell proliferation

Cell proliferation was determined measuring bromodeoxiuridine (BrdU) incorporation following the indications of the commercial assay (Roche Diagnostics, Ref. 11 647 229 001, Mannheim, Germany).

### Statistical analysis

**Fig 1:**
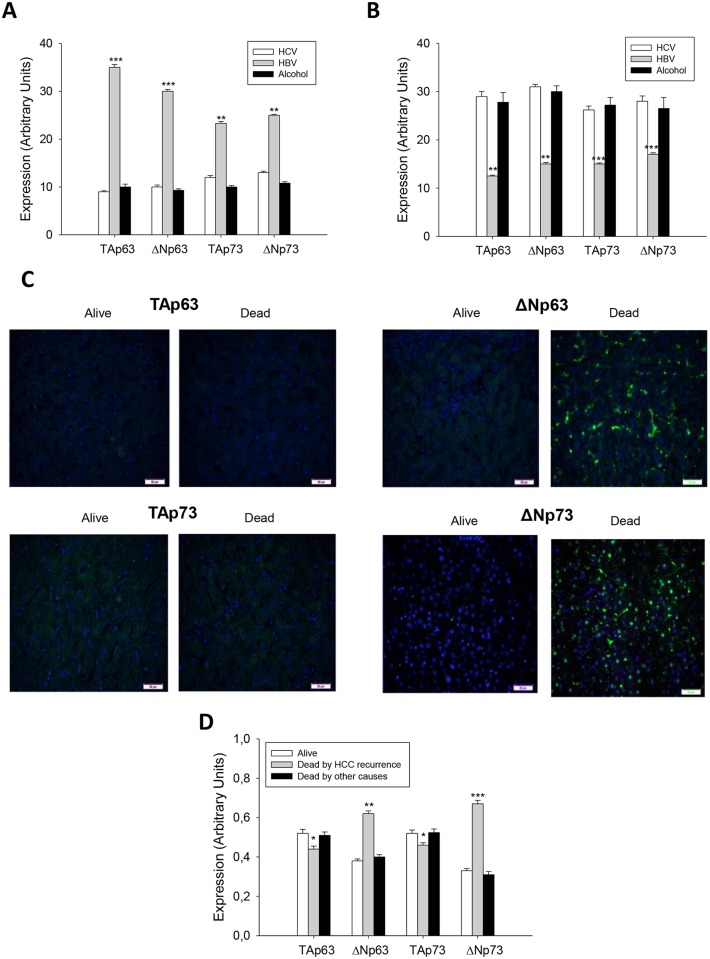
TAp63, ΔNp63, TAp73 and ΔNp73 expression in well-differentiated (A) and moderately-poorly differentiated HCC (B) in patients subject to OLT, and their correlation to survival or death as a consequence of tumor recurrence or other causes such as pneumonia, fungal sepsis, heart complications and septic shock (C). The protein expression of p53 gene family members was assessed by immunohistochemistry following the procedure described in Material and Methods. Data are expressed as mean ± SEM of the densitometry analysis. The asterisks indicate statistical significance (*p≤0.05, **p≤0.01 or ***p≤0.001) compared with their corresponding control. The images are representative of the corresponding group. Image magnification x10.

**Fig 2:**
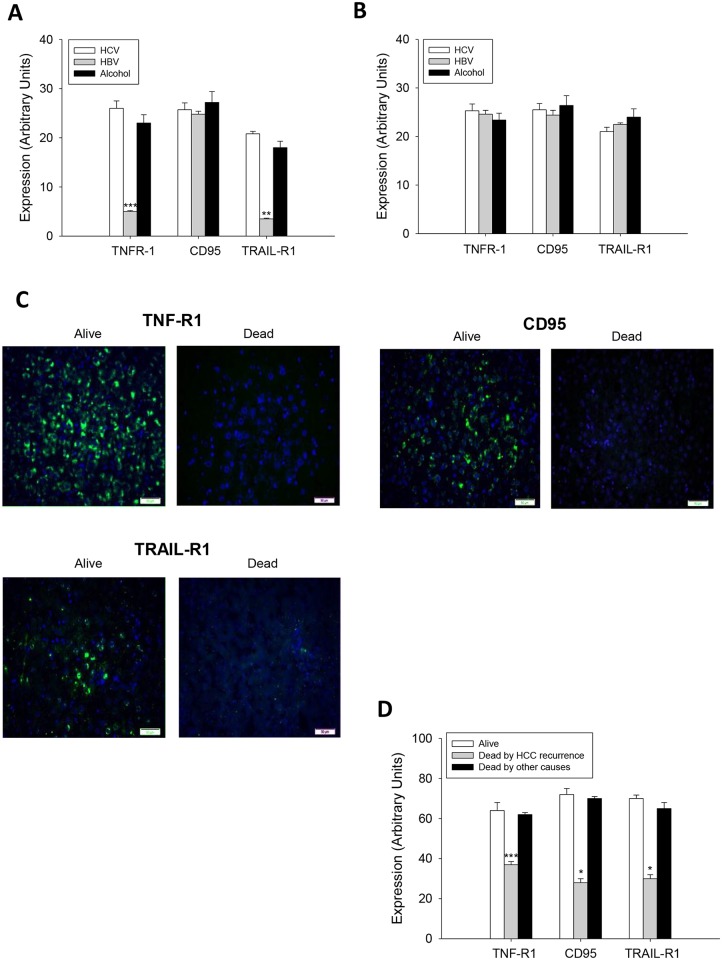
TNF-R1, CD95 and TRAIL-R1 expression in well-differentiated (A) and moderately-poorly differentiated HCC (B) in patients subject to OLT, and their correlation to survival or death as a consequence of tumor recurrence or other causes such as pneumonia, fungal sepsis, heart complications and septic shock (C). The protein expression of cell death receptors was assessed by immunohistochemistry following the procedure described in Material and Methods. Data are expressed as mean ± SEM of the densitometry analysis. The asterisks indicate statistical significance (*p≤0.05, **p≤0.01 or ***p≤0.001) compared with their corresponding control. The images are representative of the corresponding group. Image magnification x10.

Results are expressed as mean ± SE of five to ten experiments. Data were compared using ANOVA with the Least Significant Difference test as posthoc multiple comparison analysis. The comparisons of experimental groups vs their corresponding control group are shown using asterisks (*p≤0.05, **p≤0.01, ***p≤0.001). The ANOVA analysis allows the comparison among all groups in which statistical differences (p ≤ 0.05) are shown using different letter (a, b, c or d). The Pearson correlation coefficient (*r*) was used to study the correlation between the expression of p63 and p73 isoforms with the expression of cell death receptors in tumors (Figs 1D and 2D) (*p≤0.05, **p≤0.01).

## Results

### The reduced expression of TAp63 and TAp73, cell death receptors, and increased expression of ΔNp63 and ΔNp73 in HCC were related to tumor recurrence and reduced survival in patients

The expression of TAp63 and ΔNp63, as well as TAp73 and ΔNp73 isoforms were significantly increased in differentiated (Fig 1A) (p≤0.001), but reduced in moderately-poorly differentiated (Fig 1B) (p≤0.01), HCC from HBV-infected patients compared to other etiologies. Interestingly, the decreased expression of TAp63 (p≤0.05) and TAp73 (p≤0.05), and increased expression of ΔNp63 (p≤0.01) and ΔNp73 (p≤0.01) in HCC were associated with reduced recurrence-free survival when compared with patients who were alive or died by pneumonia, fungal sepsis, heart complications and septic shock (Fig 1C and 1D).

The expression of TNF-R1 and TRAIL-R1 was significantly reduced in differentiated HCC from HBV-infected patients (Fig 2A) (p≤0.01 and p≤0.01, respectively). No differences were observed on the expression of TNF-R1 (p≤0.01), CD95 (p≤0.01) and TRAIL-R1 (p≤0.01) in moderately-poorly differentiated HCC (Fig 2B). Notably, a drastic reduction on the expression of cell death receptors was observed in HCC from transplanted patients who died by tumor recurrence (Fig 2C and 2D).

**Fig 3:**
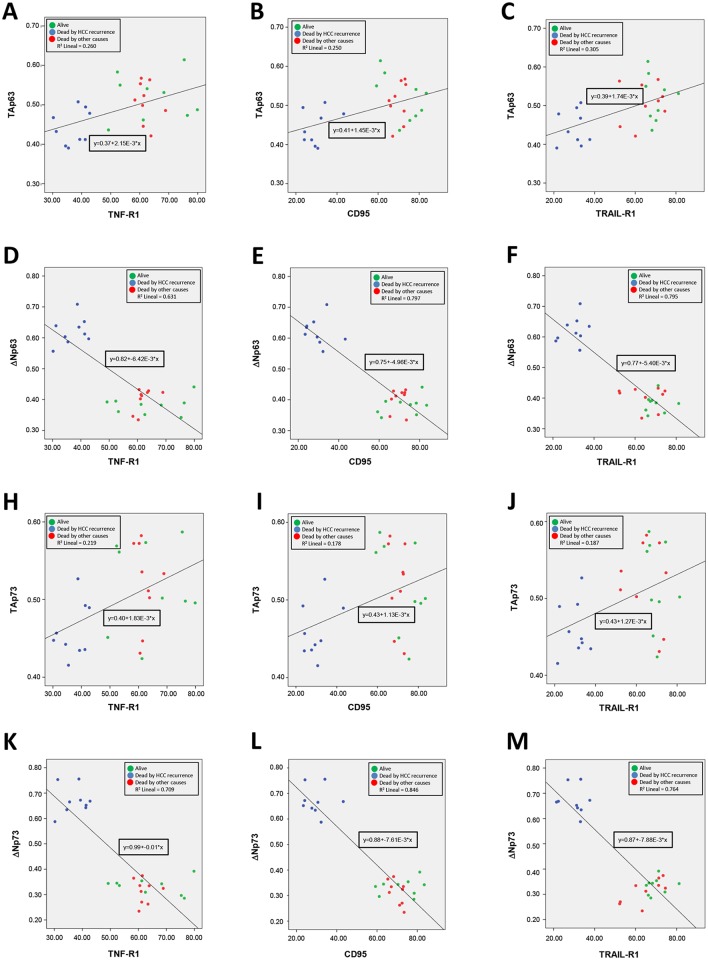
Distribution of all pairwise variables and the corresponding linear equations in the three-studied populations (alive, dead by HCC recurrence and dead by other causes). The values of R^2^ lineal of the linear equation were superior between ΔN isoforms and cell death receptors (Fig 3D-3F and 3K-3M) that those between ΔN isoforms and cell death receptors (Fig 3A-3C and 3H-3J).

The correlation of Person between the expression of p63 and p73 isoforms in alive and dead patients, as well as with the expression of cell death receptors, is presented in Table 2. The analysis showed that a positive correlation exists between TA isoform and cell death receptor expression, while a negative correlation exists between ΔN isoforms and cell death receptor expression (Table 2). The distribution of all pairwise variables and the corresponding linear equations in the three-studied population (alive, dead by HCC recurrence and dead by other causes) are shown in Fig 3. The values of R^2^ lineal of the linear equation were superior between ΔN isoforms and cell death receptors (Fig 3D–3F and 3K–3M) that those between ΔN isoforms and cell death receptors (Fig 3A–3C and 3H–3J).

**Table 2:** Values of Pearson correlation coefficient (r) and the significance of the correlation (*p*) between the expression of p63 and p73 isoforms, and cell death receptors in studied population.

	TNF-R1	CD95	TRAIL-R1
**TAp63**	r = 0.510**, *p* = 0.007	r = 0.500**, *p* = 0.008	r = 0.552**, *p* = 0.003
**ΔNp63**	r = -0.794**, *p* = 0.000	r = -0.893**, *p* = 0.000	r = -0.892**, *p* = 0.000
**TAp73**	r = 0.468*, *p* = 0.014	r = 0.422*, *p* = 0.028	r = 0.432**, *p* = 0.024
**ΔNp73**	r = -0.842**, *p* = 0.000	r = -0.920**, *p* = 0.000	r = -0.874**, *p* = 0.000

The values of Pearson correlation coefficient range from 1 and -1 (1 reflects positive linear correlation, 0 is no correlation, and -1 is negative linear correlation)

(*p≤0.05,

**p≤0.01).

### The reduced expression of TA and ΔN isoforms was related to diminished expression of cell death receptors in Hep3B vs HepG2 cells

**Fig 4:**
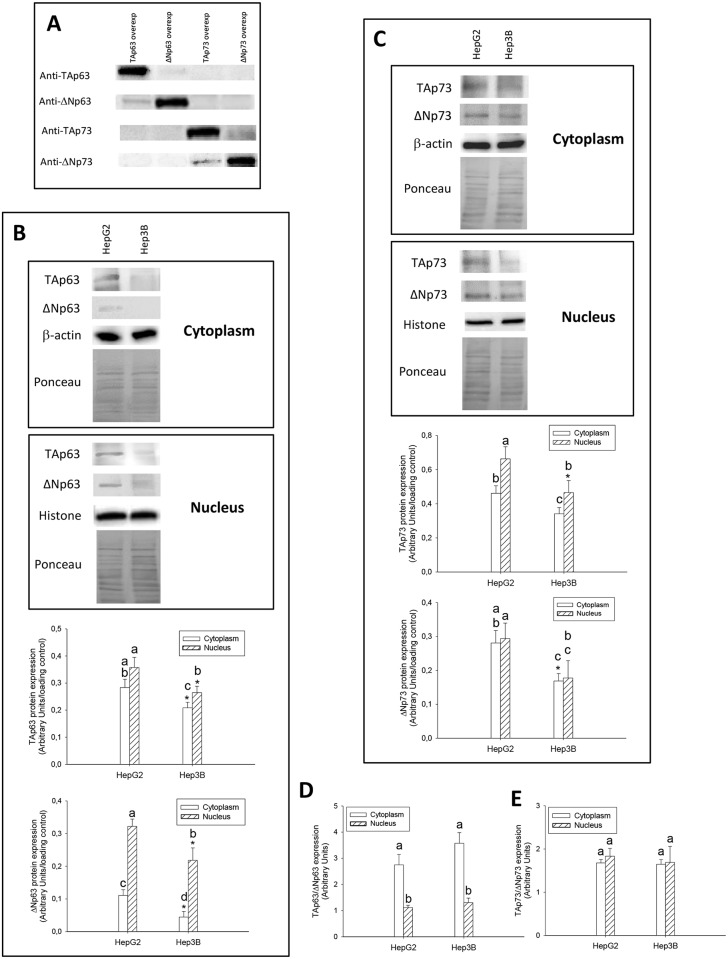
TAp63 and ΔNp63 (B), TAp73 and ΔNp73 (C) protein expression in cytoplasm and nuclear fractions, and their corresponding TAp63/ΔNp63 (D) and TAp73/ΔNp73 (E) protein ratio obtained from HepG2 and Hep3B cells. The specificity of antibodies against the different TA and ΔN p63 and p73 isoforms was assessed by western-blot analysis in transfected HepG2 using TA and ΔN p63 and p73 overexpressing plasmids (A). The protein expression was assessed by western-blot analysis following the procedure described in Material and Methods. Data are expressed as mean ± SEM of the densitometry analysis referred to the corresponding loading control (β-actin and histone H3 in cytoplasm and nuclear fractions, respectively). The ponceau staining is also included (B and C). The asterisks indicate statistical significance compared with their corresponding control group (HepG2) (*p≤0.05). The groups with different letter (a, b, c or d) were significantly different among them (p≤0.05).

**Fig 5:**
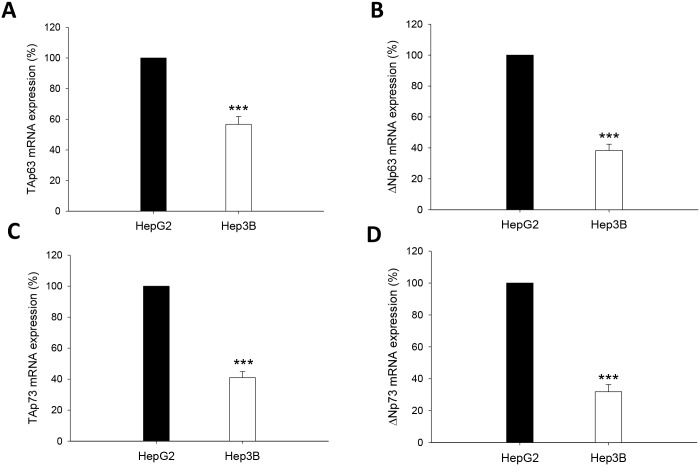
TAp63 (A), ΔNp63 (B), TAp73 (C) and ΔNp73 (D) mRNA expression from HepG2 and Hep3B cells. The mRNA expression of p53 gene family members was assessed by RT-PCR analysis following the procedure described in Material and Methods. The primers used for TAp63α, ΔNp63α, TAp73α, ΔNp73α and β-actin are detailed (E). Data are expressed as mean ± SEM referred to the corresponding constitutive control (β-actin). The asterisks indicate statistical significance compared with their corresponding group (HepG2) (***p≤0.001).

The protein specificity of antibodies against the different TA and ΔN p63 and p73 isoforms was assessed by western-blot analysis in HepG2 overexpressing p63 or p73 isoforms (Fig 4A) (p≤0.05). The expression of TAp63 and ΔNp63 (Fig 4B), and TAp73 and ΔNp73 (Fig 4C), was significantly reduced in cytoplasm and nuclear fractions from Hep3B vs HepG2 cells (p≤0.05). However, the cytoplasmic and nuclear TAp63/ΔNp63 and TAp73/ΔNp73 protein expression ratios were not significantly different between both cell lines (Fig 4D and 4E) (p≤0.05). The mRNA level of p63 and p73 isoforms was also reduced in Hep3B vs HepG2, but it appeared that the downregulation affected more ΔNp63 than TAp63 (Fig 5B vs 5A), and ΔNp73 than TAp73 (Fig 5D vs 5C) (p≤0.001).

**Fig 6:**
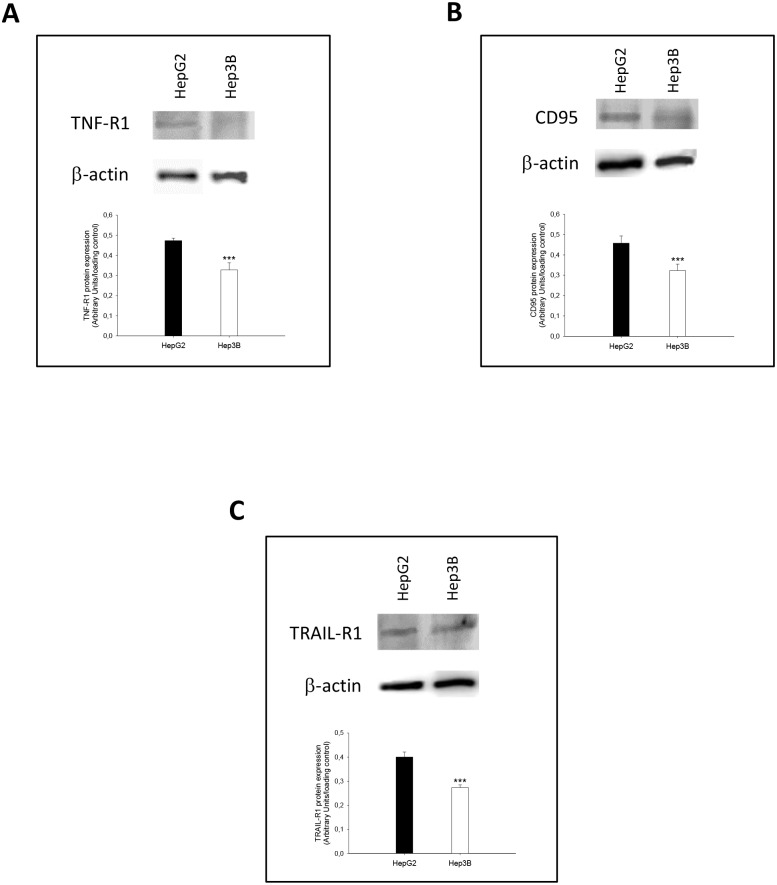
TNF-R1 (A), CD95 (B) and TRAIL-R1 (C) expression obtained from HepG2 and Hep3B. The protein expression of cell death receptors was assessed by western-blot analysis following the procedure described in Material and Methods. Data are expressed as mean ± SEM of the densitometry analysis referred to the corresponding loading control (β-actin). The asterisks indicate statistical significance compared with their corresponding group (HepG2) (***p≤0.001).

The expression of TNF-R1 (Fig 6A) (p≤0.001), CD95 (Fig 6B) (p≤0.001) and TRAIL-R1 (Fig 6C) (p≤0.001) was also reduced in Hep3B vs HepG2.

### The overexpression of TAp63α, ΔNp63α, TAp73α and ΔNp73α differentially affects the expression of cell death receptors, apoptosis, cFLIP and cell proliferation

**Fig 7:**
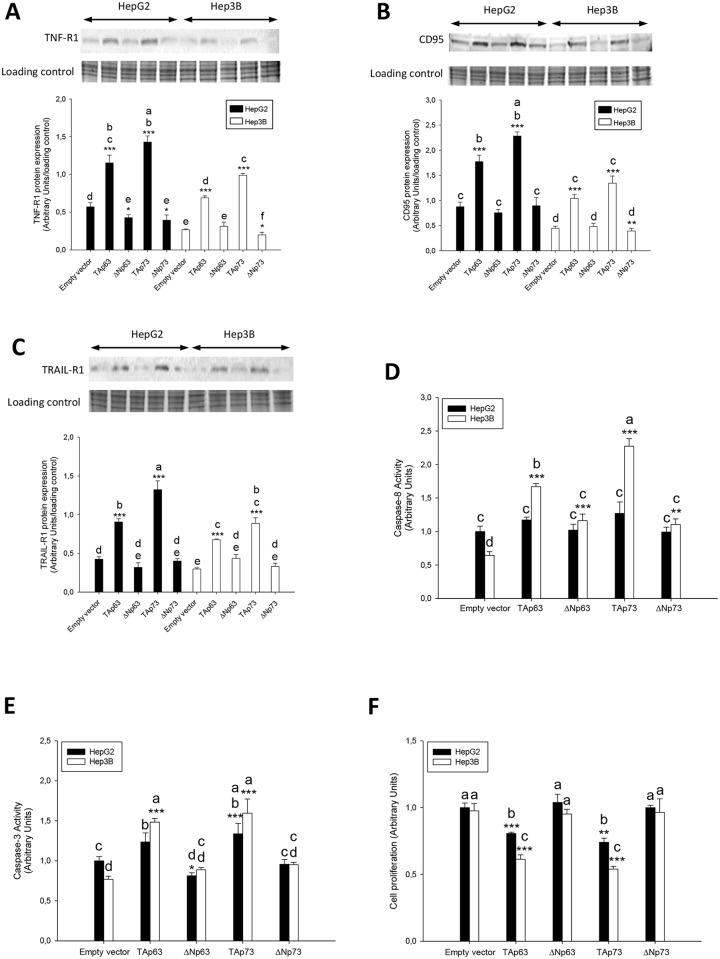
TNF-R1 (A), CD95 (B) and TRAIL-R1 (C) expression, caspase-8 (D) and caspase-3 (E) activities and cell proliferation (F) in transfected HepG2 and Hep3B cells with TAp63α, ΔNp63α, TAp73α and ΔNp73α overexpressing plasmids. The parameters were assessed 48 hours after cell transfections. The protein expression of cell death receptors was assessed by western-blot analysis following the procedure described in Material and Methods. Caspase-8 and -3 activities were determined using commercial assays described in Material and Methods. Cell proliferation was determined by BrdU incorporation administered 2 hours before cell collection following the procedure described in Material and Methods. Data are expressed as mean ± SEM either from the densitometry analysis of blots referred to the corresponding loading control (stain-free procedure) or to caspase activity referred to a calibration curve developed using recombinant purified enzyme. The asterisks indicate statistical significance (*p≤0.05, **p≤0.01 or ***p≤0.001) compared with their corresponding empty vector transfected cell line. The groups denoted by different letters (a, b, c or d) are significantly different among them (p≤0.05).

**Fig 8:**
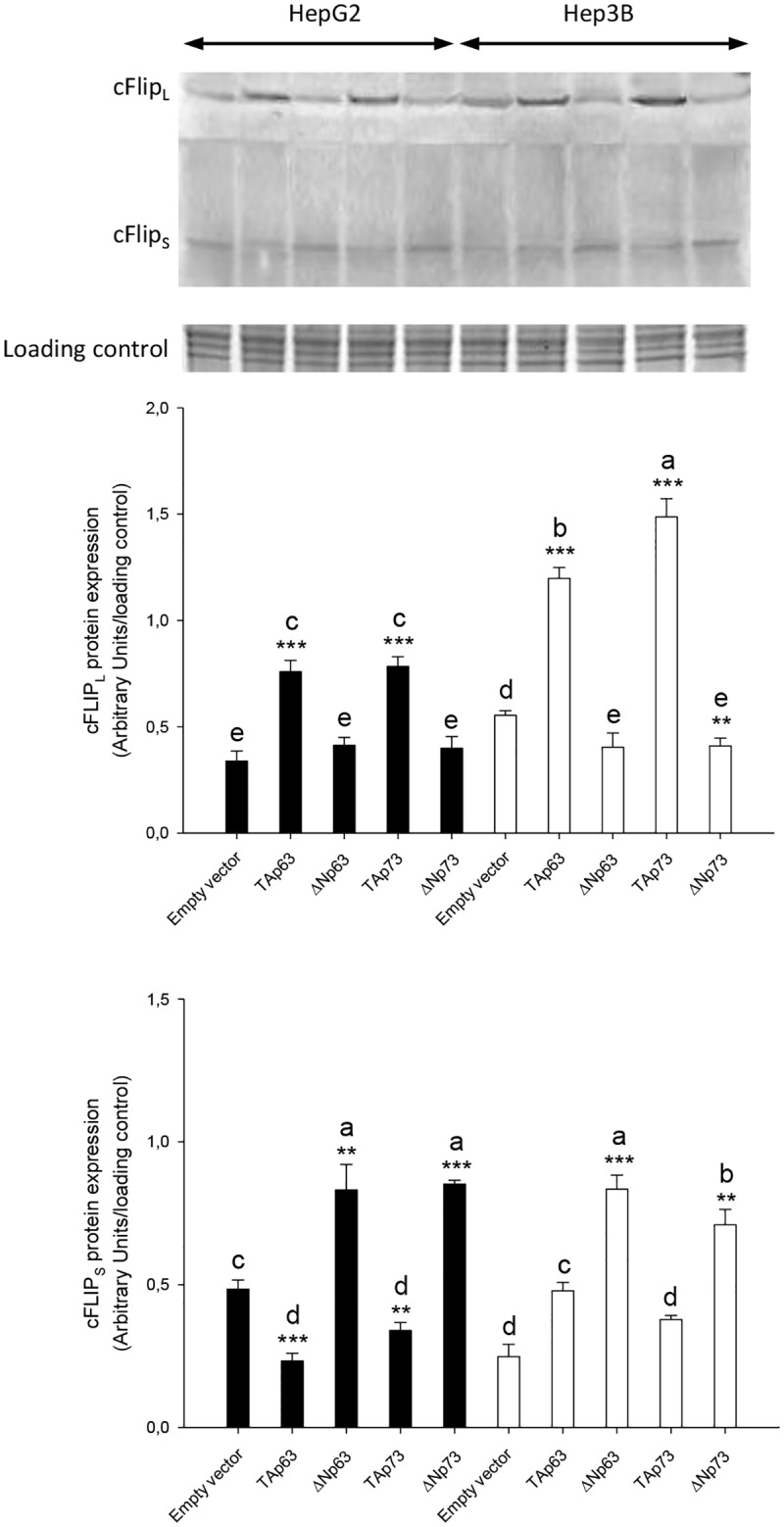
cFLIP_L_ and cFLIP_S_ expression in transfected HepG2 and Hep3B cells with TAp63α, ΔNp63α, TAp73α and ΔNp73α overexpressing plasmids. The protein expression of cFLIP was assessed by western-blot analysis following the procedure described in Material and Methods. Data are expressed as mean ± SEM either from the densitometry analysis referred to the value of stain-free procedure. The asterisks indicate statistical significance (**p≤0.01 or ***p≤0.001) compared with their corresponding empty vector transfected cell line. The groups denoted by different letters (a, b, c or d) are significantly different among them (p≤0.05).

The expression of cell death receptors (Fig 7A–7C) and caspase activation (Fig 7D and 7E) was reduced, and cFLIP_L_/cFLIP_S_ ratio (Fig 8) was increased in empty vector transfected Hep3B cells vs HepG2 cells (p≤0.001). The overexpression of TAp63 and TAp73 significantly increased the expression of cell death receptors and apoptosis in HepG2 and Hep3B (p≤0.001). Although the upregulation of cell death receptors in TAp63 and TAp73-transfected Hep3B was lower than observed in HepG2 cells, it was associated with increased caspase-8 (Fig 7D) and caspase-3 (Fig 7E) activities (p≤0.001) and cFLIP_L_ upregulation (Fig 8). It was observed a reduction of TNF-R1 expression (Fig 7A) and caspases-3 (Fig 7E) in ΔNp63-transfected HepG2 cells (p≤0.05).

TAp63 and TAp73 overexpression induced a more potent anti-proliferative effect in Hep3B (p≤0.001 and p≤0.001, respectively) than HepG2 (p≤0.001 and p≤0.01, respectively) (Fig 7F).

## Discussion

The development of HCC in the context of liver cirrhosis is associated with different co-morbidities, high pro-inflammatory component, oxidative/nitrosative stress and alternation of cell death and proliferation cycles which have been related to genetic and epigenetics alterations affecting relevant intracellular signaling related to epidermal growth factor receptor (EGFR), Ras/extracellular regulated signal ERK, phosphoinositol 3-kinase (PI3K)/ mammalian Target of Rapamycin (mTOR), epithelial-mesenchymal transition factor (c-met), Wnt, Hedgehog, and inactivation of apoptotic signaling [17]. The disruption of the p53/ARF pathway is detected in the 50% of HCC cases [1]. In fact, inactivation of p53 pathway is associated with the most aggressive S1 and S2 subclasses of HCC [5]. Only a small percentage of human tumors have been shown to harbor p63 and p73 mutations [18] being tumor development frequently associated with the differential expression of TA vs ΔN isoforms of p63 and p73 genes [19].

p63 and p73 can induce growth arrest and apoptosis in response to stress signals; two functional properties that they share with p53 [20]. A marked reduction of TAp63 expression and abnormal overexpression of ΔNp63 is found in primary bladder carcinomas [21]. The expression of TAp63 isoforms is differentially observed in HCC cell lines, however ΔNp63 isoform was only expressed in the context of p53-null cell line [22]. Nonetheless, a study involving patients with HCC failed to detect expression of ΔNp63 isoform in tumor samples [23]. The spliced isoform generated from the same promoter as TAp73 by splicing out exon 2, Ex2Δp73, as well as the one generated from second promoter or ΔNp73 have been shown to be up-regulated in different tumors (ovary, endometrium, cervix, vulva, vagina, breast, kidney, and colon) [24]. Different studies have described the expression of different variant p73 isoforms in HCC. The aberrantly spliced Ex2Δp73, but not ΔNp73, is upregulated in HCC [25, 26]. One study showed that an upregulation of TAp73 and ΔNp73 is observed in HCC, being there an association between TAp73 overexpression and loss of p53 functions [27]. In agreement with this observation, Flores et al. [19] showed that p73^+/-^ (and p63^+/-^) heterozygous animals develop thymic lymphomas, hemangiosarcomas, lung adenomas, squamous cell carcinoma and histiocytic sarcomas, whereas HCC was only observed at 15% in double heterozygous p53^+/-^:p73^+/-^ mice. In addition, TAp63 isoforms are essential for Ras-induced senescence, and that TAp63 deficiency enhances Ras-mediated oncogenesis in the context of p53 deficiency [28]. All these studies showed that TAp63 and TAp73 isoforms function as tumor suppressors by regulating cell death through p53-independent pathway. In our study, TA and ΔN isoforms of p63 and p73 were upregulated in well-differentiated HCC obtained from HBV-infected patients in comparison with HCC obtained from HCV-infected or alcoholic-related patients (Fig 1). A comparative study on the mRNA and protein expression of p63 and p73 isoforms was performed in HepG2 cells considering the presence of p53 wild type, and Hep3B cells which contains two major polypeptides of HBV surface antigen but no p53 expression. The data showed that Hep3B cells presented a significant downregulation of TAp63, ΔNp63, TAp73 and ΔNp73 expression compared with HepG2 cells being this effect more pronounced in ΔN than TA isoforms (Figs 4 and 5).

p53 regulates apoptosis through transcriptional and non-transcriptional mechanisms [8, 29]. TAp63 and TAp73 activate genes that play a role at different steps of the apoptosis program [10]. The tumor recurrence was related to reduced expression of TAp63 and TAp73, as well as an increased expression of ΔNp63 and ΔNp73 isoforms in tumors (Fig 1), being this pattern associated with reduced expression of cell death receptors (Fig 2). The expression of TA and ΔN isoforms was positively and negatively correlated to cell death receptor expression, respectively (Table 2 and Fig 3). HepG2 and Hep3B cells showed a similar TAp63/ΔNp63 (Fig 4C) and TAp73/ΔNp73 (Fig 4D) protein expression ratio in cytoplasm and nucleus, suggesting that the reduction of p63 and p73 isoforms in Hep3B may affect transcriptional and non-transcriptional-related properties of the studied isoforms, as well as the influence of p53 status on their expression as previously suggested [22].

The basal level of cell death receptors and caspase activity were significantly lower in Hep3B than HepG2 cells (Figs 6 and 7). In this conditions, the overexpression of TAp63α and TAp73α, considering the potential role of p53, increased the expression of cell death receptors (Fig 7A–7C), but being the increased caspase-8 and -3 (Fig 7D and 7E) activities more prominent in Hep3B. TAp63 and TAp73, as well as p53, have previously shown to mediate the upregulation of cell death receptors induced by DNA-damaging drugs [12]. cFLIP, the mammalian homologue of the previously described family of viral inhibitors (v-FLIPs) [30], blocks cell death receptor signaling by interfering with the recruitment and activation of caspase-8 by FADD [31]. The overexpression of cFLIP renders cells resistant to death receptor-mediated apoptosis in ovarian cancer cells [32]. The downregulation of cFLIP by cytotoxic agents has also been shown to sensitize cells to death receptor-induced apoptosis [33, 34]. cFLIP_L_, but not cFLIP_S_, can be either anti- or pro-apoptotic depending on its expression level [31, 35]. In particular, Hughes et al. [36] have recently shown that procaspase-8:cFLIP_L_ exhibits preferentially an activator activity, promoting DED-mediated procaspase-8 oligomer assembly, whereas procaspase-8:cFLIP_S_ lack activity and potently blocks procaspase-8 activation. The induction of apoptosis by nitrosative stress is associated with increased cFLIP_L_ and reduced cFLIP_S_ expression bound to CD95 in HepG2 cells [37]. In the present study, the reduced basal apoptotic activity of Hep3B vs HepG2 was related to an increased cFLIP_L_/cFLIP_S_ ratio (Figs 7 and 8). Interestingly, although TAp63α and TAp73α overexpression exerted a more potent stimulatory effect on cell death receptor expression in HepG2 than Hep3B, the latest cell lines showed an increased caspase activation that was associated with increased cFLIP_L_ supporting their pro-apoptotic role (Fig 8). The induction of apoptosis by TAp63α and TAp73α, considering the potential role of p53, was closely correlated with a reduction of cell proliferation in HCC cells (Fig 7F).

ΔNp63 has been suggested to downregulate p53 activity in response to DNA damage, and promote cancer growth in a p53-independent manner in bladder and lung cancer cells [38]. ΔNp63α negatively regulates CD95 expression and apoptosis induced by TAp63α, doxorubicin or mitoxantrone for 24 h in Hep3B [39]. ΔNp63α overexpression induced a significant downregulation in the expression of TNF-R1 and caspase-3 in HepG2 (Fig 7A–7E). In fact, the negative correlation between ΔN isoform and cell death receptor was higher than that positive correlation observed between TA isoforms and cell death receptors (Table 2 and Fig 3).

In conclusion, our study showed that the HBV-expressing Hep3B had reduced p63 and p73 (TA and ΔN isoforms) and cell death receptor expression, caspase activation, as well as increased cFLIP_L_/cFLIP_S_ ratio as compared with HepG2 cells. This pattern of expression may be relevant for the reduced survival of HCC patients subject to OLT following tumor recurrence. The *in vitro* studies involving TAp63 and TAp73 overexpression suggested that the downregulation of these longest isoforms, rather than upregulation of ΔNp63 and ΔNp73 isoforms, is more functionally relevant in terms of cell death and proliferation in Hep3B cells.

## Abbreviations

HBV: hepatitis B virus
HCC: Hepatocellular carcinoma
HCV: hepatitis C virus
OLT: orthotopic liver transplantation
